# Comparison of Early Enteral Nutrition Versus Early Parenteral Nutrition in Critically Ill Patients: A Systematic Review and Meta-Analysis

**DOI:** 10.3390/nu17010010

**Published:** 2024-12-24

**Authors:** Seung Min Baik, Mina Kim, Jae Gil Lee

**Affiliations:** 1 Department of Surgery, Ewha Womans University Mokdong Hospital, 1071 Anyangcheon-ro, Yangcheon-gu, Seoul 07985, Republic of Korea; baiksm@ewha.ac.kr; 2 Department of Nursing, Inha University Hospital, 313, Dokbae-ro, Michuhol-gu, Incheon 22188, Republic of Korea; mina4029@naver.com

**Keywords:** enteral nutrition, parenteral nutrition, critical illness, nutritional support, meta-analysis

## Abstract

Background: Nutritional support is crucial in critically ill patients to enhance recovery, reduce infections, and improve outcomes. This meta-analysis compared early enteral nutrition (EEN) and early parenteral nutrition (EPN) to evaluate their efficacy in adult critically ill patients. Methods: A systematic review of 14 studies involving 7618 patients was conducted, including randomized controlled trials, prospective cohorts, and retrospective analyses. The primary outcomes were mortality and infectious complications, while secondary outcomes included intensive care unit length of stay (ICU-LOS), hospital length of stay (H-LOS), mechanical ventilation days, and gastrointestinal (GI) complications. Results: The results showed no significant difference in mortality between EEN and EPN (OR 1.03, 95% CI 0.93–1.14). EEN reduced bloodstream infections (OR 0.73, 95% CI 0.57–0.93), ICU-LOS (MD −0.18 days, 95% CI −0.33 to −0.04), and H-LOS (MD −1.15 days, 95% CI −1.38 to −0.93). However, EEN was associated with higher GI complications, such as vomiting and diarrhea (OR 2.25, 95% CI 1.97–2.58), while mechanical ventilation days showed no significant difference. Conclusions: These findings support prioritizing EEN in critically ill patients with functional gastrointestinal systems to improve infection control and recovery while emphasizing the importance of careful monitoring to mitigate gastrointestinal complications.

## 1. Introduction

Nutritional support is a cornerstone of therapy for critically ill patients, aiming to improve clinical outcomes by mitigating metabolic derangements, reducing infection risks, and enhancing recovery. Recognizing its essential role, major guidelines recommend initiating nutritional support within 24–48 h of ICU admission in patients with stable hemodynamic status [1,2,3]. Enteral nutrition (EN) is often prioritized as the first-line approach due to its physiological benefits, including maintaining gut integrity, preserving immune function, and reducing the risk of infectious complications.

When EN is not feasible due to gastrointestinal dysfunction or contraindications, parenteral nutrition (PN) provides an alternative route for nutritional support. However, the optimal timing and indications for PN remain subjects of debate. Guidelines from the European Society for Clinical Nutrition and Metabolism (ESPEN) emphasize the early initiation of EN [1,2], highlighting its clinical advantages. In contrast, the American Society for Parenteral and Enteral Nutrition (ASPEN) guideline in 2022 acknowledges that early EN or PN may be appropriate depending on patient conditions [4], reflecting a more flexible approach.

Previous meta-analyses have yielded conflicting evidence regarding the comparative efficacy of early enteral nutrition (EEN) and early parenteral nutrition (EPN). For instance, studies by Elke et al. and Zhang et al. demonstrated that EEN reduces bloodstream infections and ICU length of stay (ICU-LOS) compared to EPN, with no significant difference in mortality rates between the two approaches [5,6]. Conversely, Harvey et al. [7] and Reignier et al. [8] reported no significant differences in mortality or clinical outcomes between EEN and EPN, complicating the interpretation of these findings. Recent reviews, such as the Cochrane review by Fuentes Padilla et al. [9] and the meta-analysis by Grillo-Ardila et al. [10], have highlighted additional discrepancies in outcomes, particularly regarding infection control and gastrointestinal complications.

Given these inconsistencies, this study aims to comprehensively evaluate EEN and EPN by synthesizing data from randomized controlled trials (RCTs) and non-randomized studies (NRSs) such as prospective cohort studies and propensity score-matched retrospective studies. By analyzing key outcomes, including mortality, infection rates, ICU-LOS, and gastrointestinal (GI) complications, this meta-analysis seeks to clarify the benefits and limitations of each nutritional approach. The findings aim to provide evidence-based recommendations for optimizing nutritional strategies in critically ill patients, addressing gaps in current knowledge, and supporting clinical decision-making.

## 2. Materials and Methods

### 2.1. Study Design

This systematic review and meta-analysis was conducted in accordance with the PRISMA (Preferred Reporting Items for Systematic Reviews and Meta-Analyses) guidelines. The study aimed to evaluate the comparative efficacy of early enteral nutrition (EEN) versus early parenteral nutrition (EPN) in critically ill adult patients, focusing on clinically relevant outcomes.

### 2.2. Eligibility Criteria

This meta-analysis included studies that evaluated critically ill adult patients (≥18 years) admitted to intensive care units (ICUs) and compared early enteral nutrition (EEN) initiated within 24–72 h of ICU admission to early parenteral nutrition (EPN) initiated in the same timeframe. Eligible studies reported on primary outcomes, including mortality (ICU and hospital) and infectious complications, or secondary outcomes, such as ICU length of stay (ICU-LOS), hospital length of stay (H-LOS), duration of mechanical ventilation (MV days), and gastrointestinal (GI) complications. Randomized controlled trials (RCTs), prospective cohort studies, and retrospective studies with case-matching or propensity score-matching designs were included. Studies were excluded if they involved patients undergoing surgical treatment for traumatic brain injury (TBI), abdominal trauma, or pancreatitis, pediatric populations, non-critically ill patients, or lacked direct comparisons between EEN and EPN. Case reports, reviews, or editorials without original data were also excluded to maintain methodological rigor and relevance.

### 2.3. Literature Search

Articles referenced in the foundational meta-analyses by Elke et al. and Zhang et al. served as a starting point for this review [5,6]. To ensure the inclusion of the most recent and relevant evidence, an updated search was conducted to identify studies published between January 2016 and July 2023. The search utilized databases including PubMed, Embase, Cochrane Library, and KoreaMed, with terms specified in Supplementary Table S1.

### 2.4. Data Extraction and Management

Two reviewers independently extracted data using a standardized extraction form. Discrepancies were resolved through discussion with a third reviewer. Extracted data included study characteristics (authors, year of publication, design, and sample size), population details (demographics and baseline characteristics), intervention specifics (timing, method, and duration of EEN and EPN), and reported outcomes (mortality, infectious complications, ICU length of stay [ICU-LOS], hospital length of stay [H-LOS], mechanical ventilation duration, and GI complications). In this analysis, infectious complications were analyzed based on how they were reported in each included study. Bloodstream infections (BSIs) were defined as blood culture-proven infections or catheter-related bloodstream infections, and vascular infections specifically referred to catheter-related infections. This rigorous process ensured consistency and accuracy in the inclusion of data for meta-analysis.

### 2.5. Risk of Bias Assessment

The Cochrane Risk of Bias (ROB) Tool was used to evaluate RCTs, assessing domains such as randomization, allocation concealment, blinding, incomplete outcome data, and selective reporting. For observational studies, the Risk of Bias in Non-Randomized Studies of Interventions (ROBINS-2) tool was applied, focusing on confounding, participant selection, intervention classification, adherence, missing data, and outcome measurement. Each study was categorized as having low, unclear, or high risk of bias. This comprehensive assessment ensured the inclusion of high-quality evidence while minimizing bias in the meta-analysis.

### 2.6. Statistical Analysis

Meta-analyses were conducted using Review Manager (RevMan) 5.4.1 software. Odds ratios (ORs) with 95% confidence intervals (CIs) were calculated for binary outcomes, and mean differences (MDs) with 95% CIs were used for continuous outcomes. Heterogeneity was assessed using the *I*^2^ statistic, with thresholds of ≤25% indicating low heterogeneity, 26–75% moderate heterogeneity, and >75% high heterogeneity. Fixed-effects models were applied for low heterogeneity, while random-effects models were used for moderate or high heterogeneity. The quality of evidence for each outcome was evaluated using the Grading of Recommendations, Assessment, Development, and Evaluations (GRADE) approach, which considered study limitations, inconsistency, indirectness, imprecision, and publication bias. The summary of findings (SoF) table was generated using GRADEpro software (gdt.gradepro.org) to provide a clear overview of evidence quality.

### 2.7. Ethical Considerations

This study was conducted as a secondary analysis of previously published data and therefore did not require ethical approval or informed consent from participants. All included studies were presumed to have followed ethical principles in line with their respective institutional and national research ethics guidelines. The meta-analysis adhered to the highest standards of research integrity and transparency by following protocols outlined in the PRISMA guidelines. Data handling was conducted with strict adherence to confidentiality and privacy principles, ensuring that no identifiable information from original studies was disclosed. The study design and execution respected the principles outlined in the Declaration of Helsinki, emphasizing the ethical use of research data to advance clinical understanding and improve patient outcomes. By exclusively analyzing data from peer-reviewed, ethically approved studies, this research ensured compliance with established ethical norms in systematic reviews and meta-analyses.

## 3. Results

**Figure 1:**
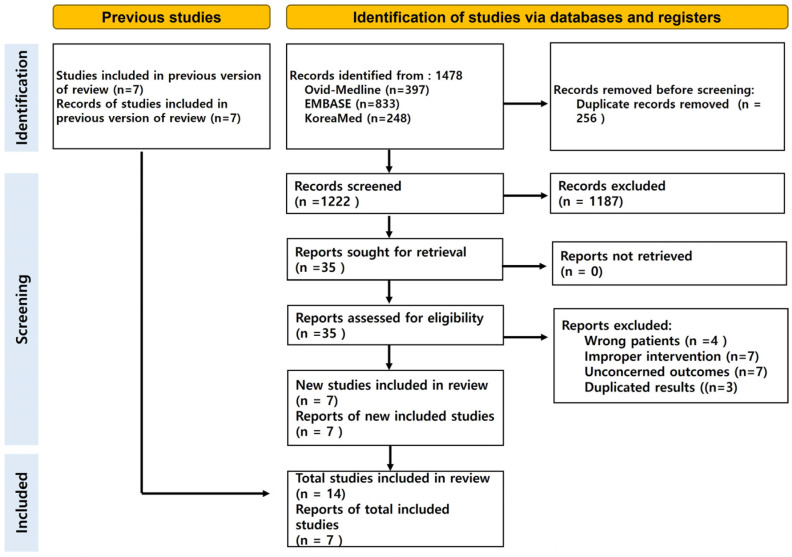
Flowchart of enrolled studies.

The systematic search identified 1478 references from databases, including Ovid-Medline (*n* = 397), Embase (*n* = 833), and KoreaMed (*n* = 248). After removing 256 duplicate records, 1222 studies were screened. From these, 35 full-text articles were assessed for eligibility, and 14 studies were included in the final analysis. Reasons for exclusion included irrelevant outcomes (*n* = 7), improper interventions (*n* = 7), wrong patient population (*n* = 4), and duplicate results (*n* = 3). Seven new studies were added to the existing body of evidence, resulting in a total of 14 studies and 7618 patients included in this review [7,8,11,12,13,14,15,16,17,18,19,20,21,22] (Figure 1). The characteristics of the included studies are summarized in Table 1, while detailed outcomes are presented in Supplementary Table S2.

**Figure 2:**
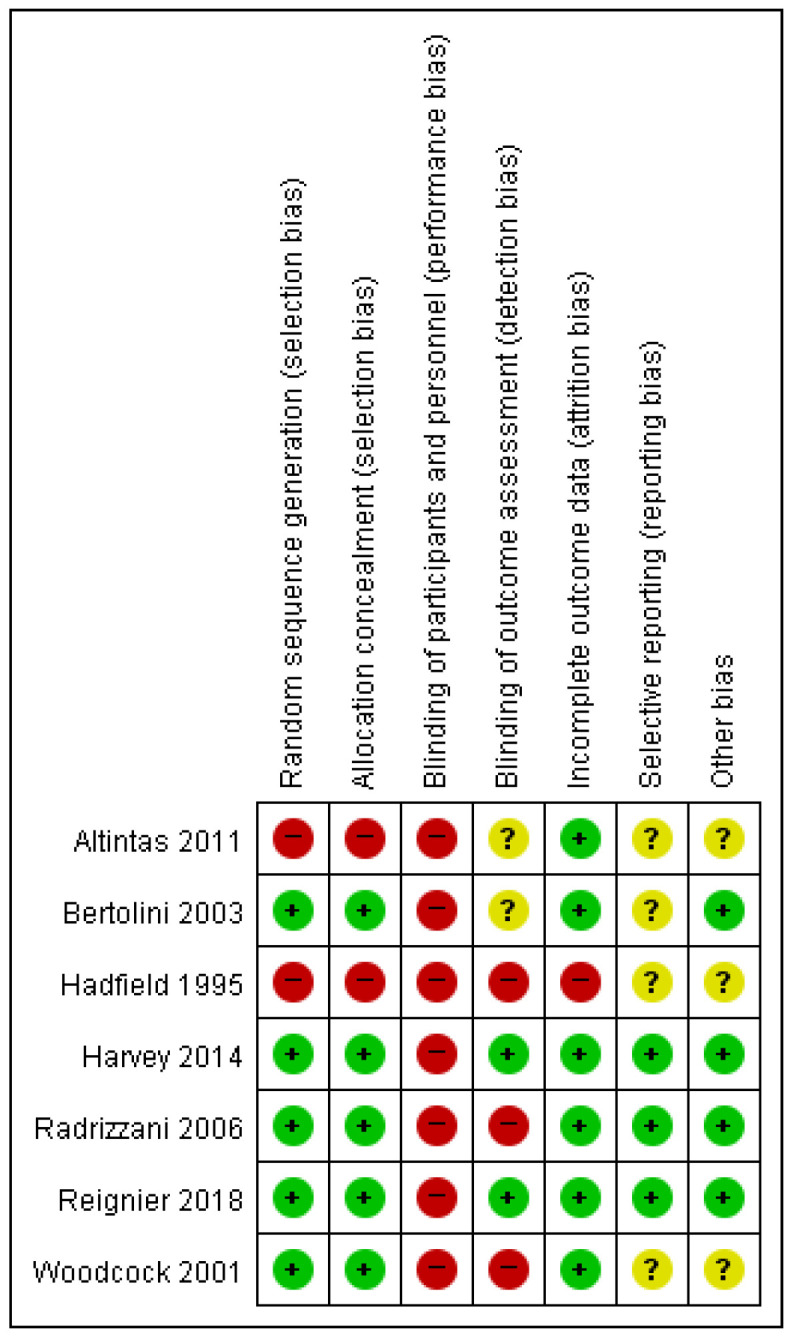
Assessment of risk of bias for RCTs using the Cochrane Risk of Bias (ROB) Tool. Green indicates low risk of bias, yellow indicates unclear risk, and red indicates high risk. Domains assessed include randomization, allocation concealment, blinding, incomplete outcome data, and selective reporting [7,8,11,12,15,18,21].

**Figure 3:**
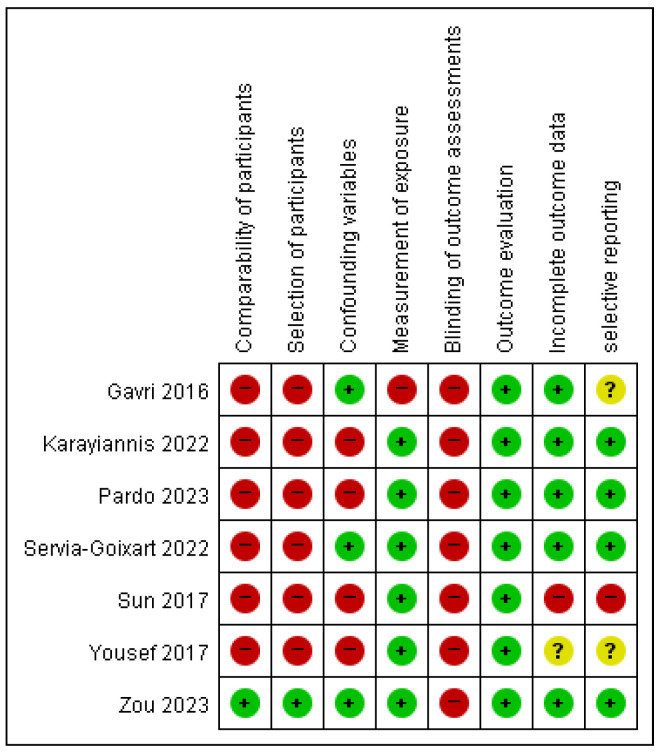
Assessment of risk of bias for non-RCTs using the ROBINS-2 Tool. Green indicates low risk, yellow indicates moderate risk, and red indicates serious or critical risk. Domains include confounding, selection of participants, classification of interventions, adherence to interventions, missing data, and measurement of outcomes [13,14,16,17,19,20,22].

The included studies varied in design (RCTs and observational studies) and population, enabling a comprehensive evaluation of clinical outcomes, such as mortality, infectious complications, length of stay (LOS), mechanical ventilation (MV) duration, and gastrointestinal (GI) complications. The risk of bias was assessed using the Cochrane Risk of Bias (ROB) tool for randomized controlled trials (RCTs) (Figure 2) and the ROBINS-II tool for observational studies (Figure 3). Heterogeneity across outcomes varied, influenced by study design, methodological quality, and patient characteristics. Mortality outcomes demonstrated moderate heterogeneity (*I*^2^ = 36% in RCTs, 58% in non-randomized studies [NRSs]), likely due to differences in patient severity and study settings. Infectious complications had low heterogeneity (*I*^2^ < 25%), while GI complications showed moderate variability (*I*^2^ = 45%), reflecting inconsistencies in definitions and reporting.

Sensitivity analyses excluding high-risk studies reduced heterogeneity for outcomes like mortality and ICU-LOS. Subgroup analyses based on illness severity and sepsis status further clarified sources of variability, emphasizing the need to minimize methodological biases in future research. The quality of evidence and the “summary of findings” (SoF) table can be found in Supplementary Figure S1.

### 3.1. Mortality

**Figure 4:**
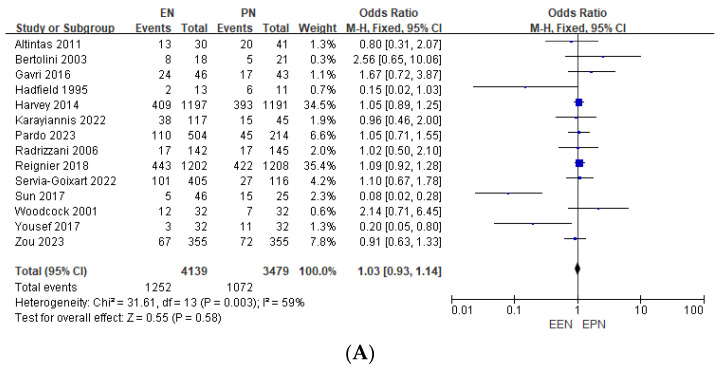

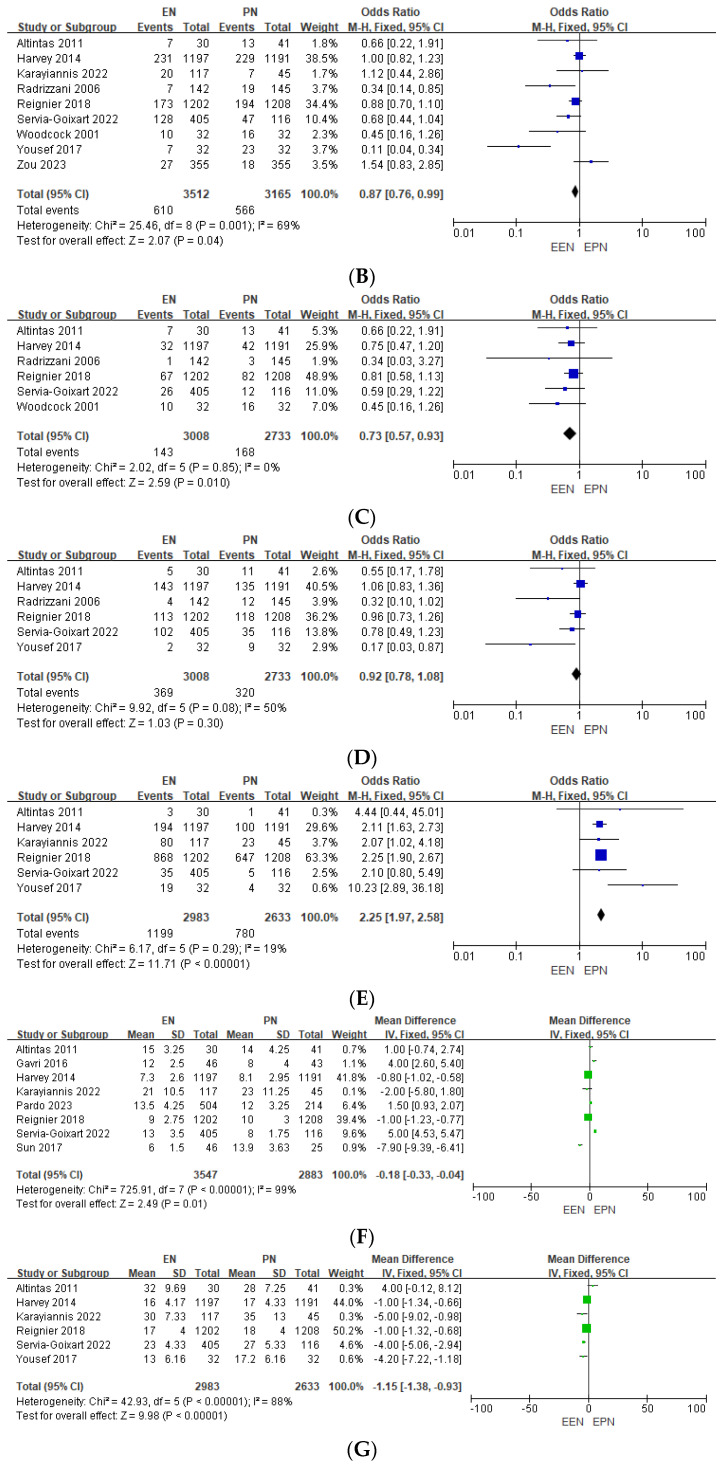

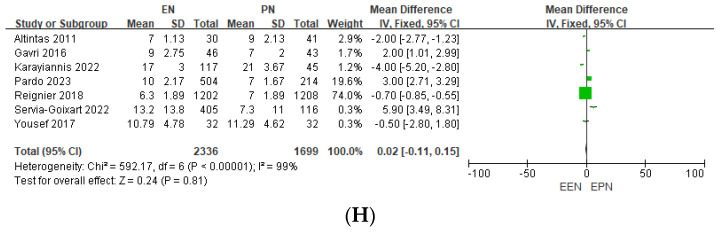
(**A**). Forest plot for mortality comparing EEN vs. EPN. (**B**). Forest plot for new infection rates comparing EEN vs. EPN. (**C**). Forest plot for new blood stream infection or bacteria comparing EEN vs. EPN. (**D**). Forest plot for new pneumonia comparing EEN vs. EPN. (**E**). Forest plot for gastrointestinal complications comparing EEN vs. EPN. (**F**). Forest plot for the length of stay in the intensive care unit comparing EEN vs. EPN. (**G**). Forest plot for the length of stay in the hospital comparing EEN vs. EPN. (**H**). Forest plot for the days of mechanical ventilation comparing EEN vs. EPN. (EN, enteral nutrition; EEN, early enteral nutrition; PN, parenteral nutrition; EPN, early parenteral nutrition) [7,8,11,12,13,14,15,16,17,18,19,20,21,22].

Seven RCTs and seven NRSs reported mortality outcomes, including ICU, hospital, 28-day, and 30-day mortality. For this analysis, all data were pooled to evaluate overall mortality. While two NRSs suggested lower mortality rates with EEN, the overall mortality difference between EEN and EPN was not statistically significant (Odds Ratio [OR] 1.03, 95% Confidence Interval [CI] 0.93–1.14, *p* = 0.58) (Figure 4A). Notably, two large-scale studies within the dataset also showed no significant difference in mortality between the two groups, likely influencing the overall pooled results and supporting the conclusion that mortality outcomes between EEN and EPN are comparable.

### 3.2. Infectious Complications

Five RCTs and four NRSs reported infectious complication frequencies. EEN was associated with a reduced risk of overall new infections (OR 0.87, 95% CI 0.76–0.99, *p* = 0.04) (Figure 4B) and bloodstream infections (OR 0.73, 95% CI 0.57–0.93, *p* = 0.01) (Figure 4C). However, no significant difference was observed in pneumonia risk between EEN and EPN (OR 0.92, 95% CI 0.78–1.08, *p* = 0.30) (Figure 4D). These findings highlight EEN’s efficacy in reducing bloodstream infections, while its effect on respiratory infections remains unclear.

### 3.3. Gastrointestinal Complications

GI complications were reported as vomiting, diarrhea, ileus, GI intolerance, and bowel ischemia (in one study). These complications were collectively analyzed as overall GI complications. EEN significantly increased the incidence of GI complications (OR 2.25, 95% CI 1.97–2.58, *p* < 0.0001) (Figure 4E). Although these events were generally self-limiting, they underscore the need for careful monitoring and individualized patient selection to optimize outcomes and minimize risks during EEN administration.

### 3.4. Length of Stay

Eight studies reported ICU-LOS, while seven studies provided data on H-LOS. Pooled analysis showed that EEN was associated with a modest reduction in ICU-LOS compared to EPN (MD −0.18 days, 95% CI −0.33 to −0.04, *p* = 0.01) (Figure 4F). Additionally, EEN significantly reduced H-LOS (MD −1.15 days, 95% CI −1.38 to −0.93, *p* < 0.001) (Figure 4G). These findings suggest that EEN may contribute to faster recovery and shorter hospitalization for critically ill patients with stable gastrointestinal function. However, the clinical significance of the ICU-LOS reduction remains modest.

### 3.5. Mechanical Ventilation Days

Seven studies reported MV duration. Some studies presented data as MV-free days, which could not be converted to MV days for analysis and were excluded. Pooled analysis showed no significant difference in MV duration between EEN and EPN groups (MD 0.02 days, 95% CI −0.11 to 0.15, *p* = 0.81) (Figure 4H). These findings suggest that EEN and EPN have comparable effects on MV duration.

### 3.6. Meta-Analysis of RCT

To address the concern regarding the inclusion of non-randomized studies (NRSs) in our analysis, we conducted a subgroup meta-analysis that included only randomized controlled trials (RCTs) (Table 2). The RCT-only meta-analysis included seven studies and revealed the following results: no significant difference in mortality between early enteral nutrition (EEN) and early parenteral nutrition (EPN) (OR 1.01, 95% CI 0.90–1.13, *p* = 0.86); a non-significant trend toward reduced infectious complications with EEN (OR 0.81, 95% CI 0.62–1.07, *p* = 0.14); a significant reduction in bloodstream infections with EEN (OR 0.76, 95% CI 0.59–1.00, *p* = 0.05); no significant difference in pneumonia rates (OR 0.90, 95% CI 0.70–1.16, *p* = 0.41); a higher incidence of gastrointestinal complications with EEN (OR 2.21, 95% CI 1.92–2.55, *p* < 0.001); a significant reduction in mechanical ventilation days with EEN (MD −1.29, 95% CI −2.56–−0.02, *p* = 0.05); a significant reduction in ICU length of stay (MD −0.83 days, 95% CI −1.18–−0.48, *p* < 0.001); and a significant reduction in hospital length of stay (MD −0.98 days, 95% CI −1.22–−0.75, *p* < 0.00).

### 3.7. Meta-Analysis for Sepsis

Four studies evaluated the effects of EEN and EPN in sepsis patients. Bertolini G, et al. [12] studied patients with severe sepsis, Radrizzani D, et al. [18] focused on non-severely septic patients, Reignier J, et al. [8] conducted a prospective study on patients in shock requiring vasopressors, and Sun JK, et al. [20] performed an observational study on surgical ICU patients with sepsis. The meta-analysis showed no significant difference in mortality (OR 1.04, 95% CI 0.89–1.22, *p* = 0.64), infection rates (OR 0.83, 95% CI 0.67–1.02, *p* = 0.08), or BSI (OR 0.79, 95% CI 0.57–1.10, *p* = 0.17). However, EEN was associated with a significantly higher risk of GI complications (OR 2.25, 95% CI 1.90–2.67, *p* < 0.001) and significantly reduced ICU-LOS (MD −1.16 days, 95% CI −1.39–−0.93, *p* < 0.001), H-LOS (MD −0.97 days, 95% CI −1.29–−0.65, *p* < 0.001), and MV days (MD −0.70 days, 95% CI −0.85–−0.55, *p* < 0.001).

The meta-analysis showed that EEN significantly reduces BSI, ICU-LOS, and H-LOS compared to EPN, with no significant differences in mortality or pneumonia, but is associated with a higher risk of GI complications, emphasizing its benefits in recovery and infection control while necessitating careful monitoring and individualized patient care.

## 4. Discussion

This systematic review and meta-analysis evaluated the comparative efficacy of EEN versus EPN in critically ill adult patients. Consistent with major guidelines, including ESPEN and ASPEN [1,2,3,4,23], the results demonstrate that EEN is associated with significant reductions in BSI, ICU-LOS, and H-LOS. However, it also increases the risk of GI complications, such as vomiting and diarrhea. Mortality outcomes were comparable between EEN and EPN, aligning with findings from large-scale studies, including those by Harvey et al. [7] and Reignier et al. [8], which showed no significant difference in overall survival.

Both ESPEN [1,2] and ASPEN guidelines [3] advocate for the initiation of EEN within 24–48 h in hemodynamically stable critically ill patients. ESPEN strongly favors EEN due to its physiological benefits, including maintenance of gut integrity and immune function. In contrast, ASPEN [4] adopts a more flexible approach, suggesting that either EEN or EPN can be initiated depending on patient conditions. The findings of this meta-analysis support ESPEN’s position by demonstrating EEN’s superiority in reducing infectious complications and recovery times. However, the increased risk of GI intolerance aligns with ASPEN’s more cautious, individualized approach.

The study builds on prior meta-analyses such as Elke et al. and Zhang et al. [5,6], and this study align with other meta-analyses, including the 2022 Cochrane review by Fuentes Padilla et al. [9] and the meta-analysis by Grillo-Ardila et al. [10]. Both studies highlighted the clinical benefits of EEN in reducing infectious complications and shortening ICU and hospital stays but demonstrated no significant differences in mortality compared to EPN. The Cochrane review focused on critically ill patients receiving EEN or EPN, presenting moderate-quality evidence favoring EEN for reducing BSI and ICU-LOS [9]. However, heterogeneity across study populations and protocols limited the generalizability of its findings. Our study incorporates additional recent data and employs standardized definitions, such as unifying vascular infections and BSI, to reduce variability and enhance the reliability of pooled outcomes. Similarly, Grillo-Ardila et al. [10] demonstrated that EEN significantly reduced infectious complication rates and expedited recovery but increased GI complications, particularly diarrhea. While Grillo-Ardila et al. reported no differences in mortality, they noted the influence of large-scale studies on pooled estimates. Consistent with these findings, our meta-analysis incorporates recent large-scale studies, including those by Harvey et al. [7] and Reignier et al. [8], further reinforcing the absence of significant mortality differences between EEN and EPN. Moreover, this study emphasizes the clinical significance of GI intolerance and supports tailored nutritional approaches to minimize complications. In addition to vomiting and diarrhea, other gastrointestinal complications, such as ileus and bowel ischemia, were identified as adverse effects of EEN. These complications varied in severity, with most being self-limiting, while rare cases, such as bowel ischemia, required clinical intervention. The mechanisms underlying these adverse events may include compromised gastrointestinal motility, ischemic injury due to hypoperfusion, or intolerance to enteral feeding protocols. Clinical management strategies, such as initiating feeding protocols at lower infusion rates, postpyloric feeding, or using prokinetic agents, may help mitigate these risks. Identifying high-risk patients through early clinical assessment and adjusting feeding strategies accordingly could further reduce GI complications while preserving the benefits of EEN.

Recent retrospective studies provide additional context to these findings. Choi et al. [24] investigated EEN in neurocritically ill patients and found it reduced infectious complications and improved neurological outcomes. This aligns with our findings, particularly the significant reduction in BSI, emphasizing EEN’s role in infection control. Similarly, Lee et al. [25] conducted a propensity score-matched analysis of EEN following emergency gastrointestinal surgery, reporting reductions in mortality and H-LOS without significant increases in GI complications. This consistency reinforces the feasibility and benefits of EEN in surgically complex patients. However, Hayashi et al. [26] evaluated a dietitian-led EEN protocol in the emergency ICU and found improved nutritional adequacy and reduced diarrhea rates compared to standard practice. This contrasts with our findings, suggesting that structured, dietitian-led interventions may mitigate GI complications associated with EEN.

The results emphasize the importance of initiating EEN in critically ill patients with functional gastrointestinal tracts to reduce infection rates and expedite recovery. However, the risk of GI complications necessitates careful patient selection and monitoring, particularly in those with borderline GI function. The comparable mortality outcomes between EEN and EPN suggest that the choice of nutritional support should be individualized based on patient-specific factors, such as illness severity, sepsis status, and tolerance to enteral feeding. Although this study did not directly evaluate healthcare costs, they remain a critical factor in the selection of nutritional strategies for critically ill patients. EEN has been shown in prior studies to potentially lower costs by reducing ICU and hospital length of stay and minimizing infectious complications. These outcomes could translate into decreased utilization of antibiotics, shorter duration of mechanical ventilation, and lower overall resource use. However, the increased risk of gastrointestinal complications with EEN may offset some of these cost benefits, especially if complications require additional interventions. Future research should focus on comprehensive cost-effectiveness analyses of EEN versus EPN, incorporating both direct medical costs and indirect costs, such as long-term functional recovery and quality of life. This is particularly important in resource-limited settings, where the cost–benefit balance may have a greater impact on clinical decision-making

This study has several limitations that should be acknowledged. First, moderate heterogeneity was observed in key outcomes, such as mortality and GI complications, due to variations in study design, patient populations, and intervention protocols. Second, many NRSs exhibited a high risk of confounding, and some RCTs showed unclear allocation concealment and performance bias, reflecting the challenges of blinding in nutritional intervention studies. Third, inconsistent reporting of outcomes, such as MV-free days versus MV days, limited the ability to perform comprehensive pooled analyses for some outcomes. Fourth, the predominance of single-center studies conducted in teaching hospitals may restrict generalizability to other healthcare settings, such as community hospitals or resource-limited environments. Lastly, the lack of long-term data on outcomes such as 90-day mortality, functional recovery, and healthcare costs limits the understanding of EEN and EPN’s extended impacts.

Despite its limitations, this study has significant strengths. Adherence to PRISMA guidelines ensured rigorous and transparent methodology. The inclusion of recent large-scale studies enhanced the robustness and applicability of findings. A comprehensive search strategy allowed for the inclusion of a diverse dataset from multiple countries and healthcare settings. Validated tools, such as the Cochrane ROB tool and ROBINS-2, provided thorough quality assessments. Sensitivity analyses addressed potential biases and validated the robustness of results. Additionally, the focus on clinically relevant outcomes, including mortality, infectious complications, and LOS, provides actionable insights that can directly inform clinical practice.

Future research should address the limitations identified in this study. High-quality, multicenter RCTs with standardized protocols are needed to reduce heterogeneity and confounding effects. Hybrid feeding strategies combining EEN and EPN could be explored to balance EEN’s benefits with reduced GI complications, especially in high-risk subgroups. Studies investigating long-term outcomes, such as 90-day mortality, functional recovery, and quality of life, are essential to understanding prolonged impacts. Research on cost-effectiveness and healthcare resource utilization is also warranted. Lastly, standardized reporting of key outcomes, such as MV days and infectious complications, should be encouraged to enable more comprehensive and reliable meta-analyses in the future.

## 5. Conclusions

This systematic review and meta-analysis highlights the comparative efficacy of early enteral nutrition (EEN) versus early parenteral nutrition (EPN) in critically ill adult patients. The findings demonstrate that EEN significantly reduces bloodstream infections, ICU length of stay, and hospital length of stay compared to EPN, aligning with major guidelines such as ESPEN and ASPEN. However, EEN is associated with an increased risk of gastrointestinal complications, including vomiting and diarrhea, which underscores the importance of careful patient selection and monitoring. Mortality outcomes and mechanical ventilation duration were comparable between EEN and EPN, further supporting the need for individualized nutritional strategies based on patient-specific factors such as illness severity, sepsis status, and gastrointestinal tolerance.

These results reinforce EEN as a preferred first-line nutritional strategy for critically ill patients with functional gastrointestinal systems, emphasizing its benefits in infection control and recovery. Nonetheless, the increased risk of gastrointestinal intolerance highlights the need for tailored approaches and multidisciplinary interventions to optimize outcomes. Future research should focus on addressing limitations such as heterogeneity and reporting variability while exploring hybrid feeding strategies and evaluating long-term outcomes to guide evidence-based nutritional practices in critical care settings.

## Figures and Tables

**Table 1:** Summary of the included studies.

	Authors	Years	Study Type	Population	Numbers of Patient			Intervention
					Total	EN	PN	
RCT	Hadfield RJ, et al. [15]	1995	RCT	Patients admitted to the Adult ICU for more than 3 d requiring nutritional support	24	13	11	EN vs. PN
	Woodcock NP, et al. [21]	2001	RCT	All patients aged 18 years or over who required adjuvant nutritional support	64	32	32	TPN via peripheral or CVC vs. EN vial NG tube or gastrostomy or jejunostmy
	Bertolini G, et al. [12]	2003	RCT	ICU patients with severe sepsis; those aged over 18 years, in a high level of care, who were judged to need artificial ventilation and nutrition for at least 4 days	39	18	21	TPN vs. EN
	Radrizzani D, et al. [18]	2006	RCT	Patients admitted nonserverly septic	287	142	145	PN vs. EN
	Altintas ND, et al. [11]	2011	RCT	All patients who needed invasive mechanical ventilation in the ICU	71	30	41	PN via central or peripheral routs vs. EN via gastric or postpyloric placement
	Harvey SE, et al. [7]	2014	RCT	Adult ICU patients expected to require nutritional support for at least 2 days, as determined by a clinician within 36 h after an unplanned ICU admission that was expected to last at least 3 days.	2388	1197	1191	Nutritional support was initiated as soon as possible after randomization (within 36 h after admission) and used exclusively for 5 days (120 h) or until transition to exclusive oral feeding, discharge from the ICU, or death.
	Reignier J, et al. [8]	2018	RCT	Adults (18 years or older); ICU patients receiving invasive mechanical ventilation (more than 48 h) and vasopressor support for shock.	2410	1202	1208	PN via CVC for at least 72 h vs. EN within 24 h after intubation.
Non-RCT	Gavri C, et al. [14]	2016	Observational	Adult patients who were admitted to the ICU for more than 96 h.	89	46	43	EN vs. PN vs. EN + PN
	Sun JK, et al. [20]	2017	Observational	Adult patients admitted to the surgical ICU with sepsis diagnosed and an ICU stay for more than 48 h	71	46	25	EN via NG or NJ tube vs. PN (contraindication of EN or could not tolerate EN for 3 days or more)
	El Rahim I. Yousef A, et al. [13]	2017	Nonrandomized	Adults who were admitted to the respiratory ICU with an ICU stay for more than 48 h and nutritional risk (NRS more than 3)	64	32	32	EN vs. PN (contraindication of EN)
	Servia-Goixart L, et al. [19]	2022	prospective observational	Adult patients (age > 18 years) needing artificial nutritional therapy for >48 h and staying in ICU for 72 h.	521	405	116	EN vs. PN
	Karayiannis D, et al. [16]	2022	Observational	Adult patients requiring MV for >48 h, projected to receive NS for at least 5 days, with COVID-19 +	162	117	45	EN (NG/ND tube) vs. PN via central line
	Zou B, et al. [22]	2023	Retrospective-propensity score matching	Adult ICU patients (>18 years)	710	355	355	EN vs. PN (PN + EN) during the first 3 days of IC
	Pardo E, et al. [17]	2023	prospective, observational study	Critically ill adult patients with an expected length of stay greater than 3 days in the ICU.	718	504	214	EEN vs. EPN

RCT, randomized controlled trial; ICU, intensive care unit; EN, enteral nutrition; EEN, early enteral nutrition; PN, parenteral nutrition; EPN, early parenteral nutrition; TPN, total parenteral nutrition; CVC, central venous catheter; NG, nasogastric; ND, nasoduodenal; h, hour(s).

**Table 2:** Summary of the meta-analysis of randomized controlled trials.

	No. of Studies	OR/MD	95% CI	*p*-Value	Heterogenity
Mortality	7	1.01	0.90–1.13	0.86	*p* = 0.18, *I*^2^ = 33%
Infectious complications	5	0.81	0.62–1.07	0.14	*p* = 0.11, *I*^2^ = 48%
Blood stream infections	5	0.76	0.59–1.00	0.05	*p* = 0.55, *I*^2^ = 0%
Pneumonia	4	0.90	0.70–1.16	0.41	*p* = 0.24, *I*^2^ = 29%
GI complications	3	2.21	1.92–2.55	<0.001	*p* = 0.77, *I*^2^ = 0%
MV days	2	−1.29	−2.56–−0.02	0.05	*p* = 0.001, *I*^2^ = 91%
ICU-LOS	3	−0.83	−1.18–−0.48	<0.001	*p* = 0.05, *I*^2^ = 67%
H-LOS	3	−0.98	−1.22–−0.75	<0.001	*p* = 0.06, *I*^2^ = 65%

No., number; OR, odds ratio; MD, mean difference; CI, confidential interval; GI, gastrointestinal; MV, mechanical ventilation; ICU, intensive care unit; LOS, length of stay; H, hospital.

## Data Availability

The data supporting the findings of this study are available from the corresponding author upon reasonable request due to ethical reason.

## Supplementary Materials

The following supporting information can be downloaded at https://www.mdpi.com/article/10.3390/nu17010010/s1: Table S1. Search terms utilized combinations of the following keywords and Medical Subject Headings (MeSH); Table S2. Summary of clinical outcomes of included studies. Figure S1. GRADE for selected studies including RCT and NRSs focused on primary outcomes.
