# Supplementary material for: Comparison of Early Enteral Nutrition Versus Early Parenteral Nutrition in Critically Ill Patients: A Systematic Review and Meta-Analysis
Source: Nutrients. 2024 Dec 24;17(1):10. doi: 10.3390/nu17010010 (PMC11723109; doi:10.3390/nu17010010)
Supplement: Supplementary file 1 [file nutrients-17-00010-s001.zip › nutrients-3388621-supplementary.pdf]

Supplementary Table S1. Search terms utilized combinations of the following keywords and Medical Subject Headings (MeSH)

Patients	("Critical Illness"[MH]) OR ("Sepsis"[MH]) OR ("Shock, Septic"[MH]) OR ("Critical illnesses"[ALL] or "Critically ill"[ALL] or "ICU"[ALL] or "intensive care"[ALL] or "sepsis"[ALL] or "septic shock"[ALL])
Interventions	("Enteral Nutrition"[MH]) or ("Enteral nutrition"[ALL] or "Enteral Feeding"[ALL] or "tube Feeding"[ALL]), ("Parenteral Nutrition"[MH]) or ("Parenteral nutrition"[ALL] or "Parenteral Feeding"[ALL] or "Intravenous Feeding"[ALL])

Supplementary Table S2. Summary of clinical outcomes of included studies.

Study	Group	Total No.	Mortality (n)		Infection (n)	BSI/vascular (n)	Pneumonia (n)	ICU – LOS Mean (SD)	MV (d) Mean (SD)	H – LOS Mean (SD)	GI complications (n)	Organ dysfunction (n)
Hadfield 1995	EN	13	Overall	2								
	PN	11	Overall	6								
Woodcock 2001	EN	32	Overall	12	10	0						
	PN	32	Overall	7	16	5						
Bertolini 2003	EN	18	28 day	8								
	PN	21	28 day	5								
Radrizzani 2006	EN	142	28 day	17	7	1	4					45
	PN	145	28 day	17	19	3	12					56
Altintas 2011	EN	30	Hospital	13	7	2	5	15 (3.25)	7 (1.13)	32 (9.69)	3	
	PN	41	Hospital	20	13	4	11	14 (4.25)	9 (2.13)	28 (7.25)	1	
Harvey 2014	EN	1197	30 day	409	231	32	143	7.3 (2.6)		16 (4.17)	194	
	PN	1191	30 day	393	229	42	135	8.1 (2.95)		17 (4.33)	100	
Reignier 2018	EN	1202	28 day	443	173	67	113	9 (2.75)	6.3 (1.89)	17 (4)	868	
	PN	1208	28 day	422	194	82	118	10 (3)	7 (1.89)	18 (4)	647	
Gavri 2016	EN	46	ICU	24				12 (2.5)	9 (2.75)			
	PN	43	ICU	17				8 (4)	7 (2)			
Sun 2017	EN	46	28 day	5				6 (1.5)				10
	PN	25	28 day	15				13.9 (3.63)				15
El Rahim I. Yousef 2017	EN	32	Overall	3	7		2		10.79 (4.78)	13 (6.16)	19	
	PN	32	Overall	11	23		9		11.29 (4.62)	17.2 (6.16)	4	
Servia-Goixart 2022	EN	405	28 day	101	128	26	102	13 (3.5)	13.2 (13.8)	25 (4.33)	35	
	PN	116	28 day	27	47	12	35	8 (1.75)	7.3 (11)	27 (5.33)	5	
Karayiannis 2022	EN	117	30 day	28	20			21 (10.5)	17 (3)	30 (7.33)	80	
	PN	45	30 day	15	7			23 (11.25)	21 (3.67)	35 (13)	23	
Zou 2023	EN	355	28 day	67	27							
	PN	355	28 day	72	18							
Pardo 2023	EN	504	28 day	110				13.5 (4.25)	10 (2.17)			
	PN	214	28 day	45				12 (3.25)	7 (1.67)			

No, numbers; EN, enteral nutrition; PN, parenteral nutrition; BSI, blood stream infection; ICU, intensive care unit; LOS, length of stay; H, hospital; GI, gastrointestinal;

Supplementary Figure S1. GRADE for selected studies including RCT and NRSs focused on primary outcomes

Author(s):
Question: EEN compared to EPN for ICU
Setting: RCT & NRSs
Bibliography:

Certainty assessment							№ of patients		Effect		Certainty	Importance
№ of studies	Study design	Risk of bias	Inconsistency	Indirectness	Imprecision	Other considerations	EEN	EPN	Relative (95% CI)	Absolute (95% CI)		
**Mortality**												
7	randomised trials	not serious	not serious	not serious	not serious	none	904/2634 (34.3%)	870/2649 (32.8%)	**OR 1.07** (0.95 to 1.20)	**15 more per 1,000** (from 11 fewer to 41 more)	⊕⊕⊕⊕ High	CRITICAL
**Infection**												
5	randomised trials	not serious	not serious	not serious	not serious	none	428/2603 (16.4%)	471/2617 (18.0%)	**OR 0.90** (0.78 to 1.04)	**15 fewer per 1,000** (from 34 fewer to 6 more)	⊕⊕⊕⊕ High	CRITICAL
**BSI**												
5	randomised trials	not serious	not serious	not serious	not serious	none	102/2603 (3.9%)	136/2617 (5.2%)	**OR 0.76** (0.59 to 1.00)	**12 fewer per 1,000** (from 21 fewer to 0 fewer)	⊕⊕⊕⊕ High	CRITICAL
**Pneumonia**												
4	randomised trials	not serious	not serious	not serious	not serious	none	265/2571 (10.3%)	286/2585 (11.1%)	**OR 0.90** (0.70 to 1.16)	**10 fewer per 1,000** (from 31 fewer to 15 more)	⊕⊕⊕⊕ High	CRITICAL
**Mortality**												
7	non-randomised studies	not serious	serious	not serious	not serious	none	338/1505 (22.5%)	202/830 (24.3%)	**OR 0.74** (0.44 to 1.15)	**51 fewer per 1,000** (from 119 fewer to 27 more)	⊕◯◯◯ Very low	CRITICAL
**Infeciton**												
4	non-randomised studies	not serious	serious	not serious	not serious	none	182/909 (20.0%)	95/548 (17.3%)	**OR 0.66** (0.28 to 1.54)	**52 fewer per 1,000** (from 118 fewer to 71 more)	⊕◯◯◯ Very low	CRITICAL
**BSI**												
1	non-randomised studies	not serious	not serious	not serious	not serious	none	26/405 (6.4%)	12/116 (10.3%)	**OR 0.59** (0.29 to 1.22)	**40 fewer per 1,000** (from 71 fewer to 20 more)	⊕⊕◯◯ Low	CRITICAL
**Pneumonia**												
2	non-randomised studies	not serious	serious	not serious	not serious	none	104/437 (23.8%)	44/148 (29.7%)	**OR 0.45** (0.11 to 1.89)	**137 fewer per 1,000** (from 253 fewer to 147 more)	⊕◯◯◯ Very low	CRITICAL

CI: confidence interval; **OR:** odds ratio
